# Genito-Urinary and Syphilitic Diseases

**Published:** 1898-05

**Authors:** Byron H. Daggett

**Affiliations:** Buffalo, N. Y.


### GENITO-URINARY AND SYPHILITIC DISEASES.

Conducted by BYRON H. DAGGETT, M. D., Buffalo, N. Y.

### NEISSER'S METHOD OF TREATING GONORRHEA.

PROFESSOR NEISSER, of Breslau, states (*Dermat. Centralbl.*) that even the best method of treating gonorrhea proves unsuccessful, unless a remedy be employed which will destroy the gonococcus without giving rise to changes in the mucous membrane and increase of the inflammatory process. Of the remedies that he has tried, he gives the first place to protargol, a new organic silver compound, which has the advantage of not being precipitated by albumin, sodium chloride or alkalies, of being far more penetrating than other silver preparations and of being practically free from irritation and, therefore, available in the earliest stages of the disease. It is, therefore, adapted for prolonged injections, the solution being kept in the urethra for thirty minutes, and the patient being spared the trouble of repeated injections. It is Neisser's custom to resort at once to injections of protargol solution (¼ to 1 per cent.) as soon as the presence of gonococci has been determined.

During the first few days three injections are given daily, the fluid being retained in the urethra five minutes during two of the injections and thirty minutes during the other. Later, one prolonged injection is sufficient. In view of the fact that few physicians are able to institute examinations for gonococci, the author thinks it important that this treatment should be kept up for a sufficient length of time, so as to insure a radical cure.

---

FUNCTIONAL ALBUMINURIA.

YOUNG, J. C., Newark, N. J., (*Medical Examiner*, July, 1897,) M. B. Life Co., says: Following the publication of Bright's researches in 1827, the presence of albumin in the urine was regarded as a grave omen, indicating almost certain death at no very remote period. A few years of observation were followed by a reaction and the belief that albumin might be found in the urine under conditions compatible with perfect health was accepted. There were writers who maintained that albumin is a physiological ingredient of the urine. Albuminuria exists without endangering life or impairing the integrity of the kidneys, yet it is not physiological.

Charcot proposed three theories to account for albumin in the urine: (1) The hematogenous theory—that albuminuria was due to preliminary changes in the composition of the blood. (2) The mechanical theory—vascular changes in the kidney. (3) The degenerate theory—due to an anatomical modification of the epithelia and glandular tissue of the kidney.

One and two include all cases of functional albuminuria, and, as the hematogenous is associated or connected with the mechanical class, we may regard functional albuminuria as due to vascular changes and as explainable by the mechanical theory.

The kidney is abundantly supplied with blood by the large-sized renal artery, from which large branches pass outward as the arteriæ propriæ renales, through the columns of Bartini, form loops across the bases of the pyramids, and these give off the interlobular arteries to the cortex and the vasæ rectæ to the pyramids. These vessels give off branches, the afferent vessels, which pierce the capsule of the Malpighian body and form a plexus—the glomerulus, which is directly concerned in the secretion of albumin.

From the glomeruli arise the radicles of the renal veins, which are still filled with arterial blood. These radicles unite and form the vas efferens, which is much smaller than the corresponding artery, a condition which favors renal congestion. After passing through the capsule of Bowman, the vas efferens forms a second plexus, which surrounds the convoluted tubules. These secondary plexuses of veins form the renal venous system. The branches of the secondary plexuses unite and form the renal vein. The convoluted tubules make a dilated sac or Bowman's capsule; becoming attenuated they form the descending limbs of Henle's loops and then pass back to the cortex as the ascending limbs, becoming again convoluted and irregular tubules and emptying into the collecting tubules and thence transmit their contents to the pelvis. The epithelium is cuboidal or columnar in the convolutions and flat in Henle's loops. There are small rod-like bodies perpendicular to the lumen of the tubule situated at the deeper layers of the epithelium, loosely attached to the connective tissue, forming the septa of a reticulated structure of living protoplasm, shown by Heitzmann, Strickler and Millard to be really a formation of living matter.

*Physiology.*—It is generally conceded that the watery elements of the urine are secreted by the glomerulus, facilitated by the large volume of its vessels the thinness of their walls and the smaller calibers of the efferent vessels. The equilibrium is maintained by increased or diminished secretion of water, according to the degree of blood pressure. If the equilibrium be overcome, damage may result to the organ. The separation of the watery elements of the urine is not simply a physical phenomena, but is largely performed by the epithelia of the glomerulus.

Overbeck showed that compressing the renal artery caused suppression of the urine; relaxing the compression the circulation was at once restored, yet the urinary secretion did not appear for a half to three-quarters of an hour, owing probably to the disturbed function of the epithelia from ligation of the renal artery. Ligate the renal vein and the same result follows. Anoxemia is produced in both cases. That extractive matter of the urine, urea and uric acid, and the like, are formed chiefly in the epithelium of the convoluted tubulesis convincingly confirmed by experimentation. It is demonstrated by experiment that kidney action is necessary for the synthetical production of hippuric acid. The water of the urine is secreted by the glomerulus, the extractives are partly separated and chiefly secreted by the epithelium of the tubules.

A temporary condition of anoxemia, whether due to either arterial or venous obstruction, induces albuminuria, through diminished cell activity and vitality. The results of experiments performed upon healthy kidneys prove that albumin is secreted by the epithelial cells of the glomeruli, in the capsule of Bowman, and that retardation of the blood current through the vascular plexus of the glomeruli is an essential condition ; also that anoxemia of the blood current of the tuft causes albuminuria.

---

### CLINICAL REMARKS ON SOME SUPPURATIONS OF THE URINARY APPARATUS.

REGINALD HARRISON (*Medical Record*, July 3, 1897,) says: "The most common as well as the farthest-reaching cause of suppuration of the urinary organs is gonorrhea. The recent advances made in the bacteriology of this subject have opened the way to progressive methods of treatment. The investigations of Guyon, Janet, Halle and others have demonstrated the life history and development of these microörganisms. We learn that chronic specific suppuration is a far more extensive malady than it is generally supposed to be. This explains the difficulty in many cases experienced in promptly relieving this condition. So long as gonorrhea is confined to the anterior urethra the problem is not a difficult one.

When the bladder becomes infected it is difficult to prevent urethral recrudescence, hence the disease may be almost indefinitely prolonged. Invasion of the bladder is not uncommon, and its symptoms may be subacute, so that attention is not drawn to this complication. The prostate usually shows active manifestations when invaded. The mucous membrane of the bladder is singularly insensitive to bacterial invasion. In the bladder, during the night, the conditions are favorable for development of bacteria, which probably accounts for the matutinal suppuration exuded by the urethra. Ureteral and renal invasion are rare, but it may occur and cause pyelitis and ureteral stricture. Suspected infection of the bladder with microorganisms should be a matter of proof. For this purpose obtain a specimen of urine from the bladder without contamination by urethral germs. Vesical urine may be procured by flushing the canal with the first flow, and collecting specimens from the remainder of the output. A better way is to obtain a specimen by catheter, as directed by the Clinical Research Association. In cases of gonorrhea, examine the vesical urine for gonococci.

If gonococci are found in the discharges or in the vesical urine there is possibility of transference of the infection. If the bladder be infected, the treatment should include flushing the urinary viscus and tract with antiseptics after the measure proposed by Dr. Janet, with some modifications. The object is arrived at in the following manner: "My apparatus consists of an ordinary hydrostatic bladder tank, holding about one pint of water and fitted with a nozzle to which a No. 8 Jacques rubber catheter is attached. The tank is elevated about six feet from the ground, and is filled with warm water containing thirty minims of Condy's fluid to a pint of the latter. For lubricating the catheter I use carbolized vaseline.

"The patient, having emptied his bladder spontaneously, is placed in the recumbent position and the catheter is then passed. Before the nozzle of the irrigator is connected with the catheter as it lies in the bladder, the fluid is allowed to run off for a few seconds, so as to insure that there is no free air in the tube of the apparatus. Then the connection is made, and the fluid is allowed to flow into the bladder by degrees, until the patient is conscious of feeling distention. I generally use from twelve to sixteen ounces for this purpose, allowing it to enter the bladder in jets of about three or four ounces at a time. In this way the feeling of any sudden or extreme fulness is avoided, and the entire area of the mucous membrane of the bladder is unfolded and opened out and thus comes in contact with the permanganate solution. When a sufficient degree of bladder distention is obtained, the catheter is slowly removed, care being taken not to allow the fluid to escape. I then usually lightly palpate the bladder above the pubes with the hand before the patient stands up. This he should then do, and proceed to empty his bladder of its contents by his natural efforts. Thus not only is the bladder washed out, but the whole urethra is flushed in a manner that is impossible with any other artificial method. As the patient is voiding the contents of his bladder, it is well to direct him suddenly to interrupt the outflow once or twice by pressure with the finger on the penile urethra. In this way the lacunæ of the canal are also distended and flushed by the irrigating fluid. This completes the process, which may be repeated once or twice in the twenty-four hours, until the urine and the urethral mucus are found free from organisms. Most patients, after proper instruction, will be able to carry out all these details. On the conclusion of each irrigation the patient should rest for a time in the recumbent position."

The abstractor proposed a method of flushing the urinary tract and bladder, without a catheter and without force, in an article read July 15, 1892, and subsequently printed in the BUFFALO MEDICAL JOURNAL. Briefly, this procedure consists of the following requirements: (1) posture and flexion of the body; (2) steady dilatation of the urethra by the continuous presence of a hot saline solution. This is accomplished by means of a short double canula; the outflow is smaller than the intake, the canula being connected with a fountain fifteen or twenty inches higher than the pelvis. This method of procedure incites a vermicular, a retroperistaltic action. Civiale, McNamara and Ott have alluded to this phenomenon without applying it to practical use.

Permanganate of potash in large dilution produces good results as a flushing agent. Half a drachm of Condy's fluid, increased to a drachm, to the pint of water, neutral sulphate of quinine, a grain to the ounce, may be used. Nitrate of silver, one-sixteenth grain to the ounce, and perchloride of mercury, one part in 10,000, are valuable remedies. The mercury sometimes produces great pain, which may be relieved by refilling the bladder with a strained solution of the albumin of an egg. A weak solution of sodium chloride will relieve the irritation following the use of nitrate of silver. These remedies are to be used with great caution when irrigating the bladder. Diluted milk may be used in place of the egg albumin. Borolyptol, hydrozone, glycozone, hydrate chloral, one part to two of biborate of soda, a teaspoonful to the pint of water, campho-phenique, ichthyol with glycerine, chlorinated soda and methylene blue, are valuable irrigants in cystitis and urethritis.

Internal remedies are valuable adjuncts in the treatment of bladder and urethral disease. The vegetable oils, sandal wood, copaiba, cubebs, together with kova kova, triticum, saw palm and others.

Eucalyptol, wintergreen, boric acid and salol are administered to render the urine sterile. The late Dr. Palmer, of Louisville, demonstrated that boric acid, in ten to fifteen grain doses, usually inhibited urethral rigors and fever. Borocitrate of magnesia has a remarkable effect in sterilising and clearing purulent urine. Dose is a teaspoonful. Benzoate of sodium and salicylate of sodium āā fifteen grains to a dose in combination and given three times daily, in an ounce of chloroform water, and hyposulphite of sodium in fifteen grain doses are valuable remedies for clearing purulent urine. The reaction of the urine is the chief guide in urinary therapeusis.

